# Comparing blood pressure measurements between sitting in chairs and sitting on the floor

**DOI:** 10.1186/s40885-024-00273-w

**Published:** 2024-07-01

**Authors:** Byung Sik Kim, Young-Hyo Lim, Woohyeun Kim, Hyungdon Kook, Jeong-Hun Shin, Yonggu Lee, Ran Heo, Hyun-Jin Kim, Jinho Shin

**Affiliations:** 1 Division of Cardiology, Department of Internal Medicine, Hanyang University Guri Hospital, Guri, South Korea; 2 Division of Cardiology, Department of Internal Medicine, Hanyang University Medical Center, Hanyang University College of Medicine, 222, Wangsimni-Ro, Sungdong-Gu, Seoul, 04763 South Korea

**Keywords:** Blood pressure, Hypertension, Blood pressure determination

## Abstract

**Background:**

The current standard approach to measuring home blood pressure (BP) involves taking measurements while sitting in a chair. In cultures where floor sitting is common, including Korea, assessing BP while sitting on the floor would be more feasible. However, there is still a lack of research investigating whether BP measurements obtained while seated in a chair and while sitting on the floor can be regarded as interchangeable. The aim of the study was to evaluate whether there is a difference between BP measurements taken while sitting in a chair and while sitting on the floor in a Korean adult.

**Methods:**

Among the participants who visited for evaluation of pulse wave velocity, a total of 116 participants who agreed to participate in the study were randomly selected. All subjects rested for 5 min, and BP measurements were taken at 1-min intervals according to a randomly assigned order of standard method (chair-sitting) and BP in a seated on the floor (floor-sitting).

**Results:**

Of the 116 participants, the median age was 68 (with an interquartile range of 59 to 75), and 82% were men. There were no significant differences in systolic BP (SBP, 129.1 ± 17.8 mmHg in chair-sitting and 130.1 ± 18.9 mmHg in floor-sitting, *P* = 0.228) and diastolic BP (DBP, 73.9 ± 11.4 mmHg in chair-sitting and 73.7 ± 11.4 mmHg in floor-sitting, *P* = 0.839) between the two positions. In addition, there was a high level of agreement between BP measurements taken in the two positions (intraclass correlation coefficients: 0.882 for SBP and 0.890 for DBP).

**Conclusion:**

These findings provide important insights into securing the reliability of home BP measurements through the commonly practiced floor-sitting posture in cultures where floor sitting is common. Furthermore, this could serve as substantial evidence for providing specific home BP measurement guidelines to patients who adhere to a floor-sitting lifestyle.

**Supplementary Information:**

The online version contains supplementary material available at 10.1186/s40885-024-00273-w.

## Introduction

Accurate measurement of blood pressure (BP) is a crucial aspect of diagnosing and treating hypertension [1]. BP is dynamic, fluctuating continuously and susceptible to influences from various factors like the environment, emotions, and circadian changes [2, 3]. Depending solely on a single office BP measurement may lead to inaccurate diagnoses and unnecessary treatments for hypertension. Therefore, current hypertension guidelines emphasize the importance of repetitive office BP measurements and out-of-office BP measurements for accurate diagnosis and appropriate treatment of hypertension [2, 4].

When considering out-of-office BP measurements, ambulatory blood pressure monitoring (ABPM) is the first choice as it offers a more comprehensive assessment of BP, including phenotypes, circadian patterns, and variability. However, home blood pressure monitoring (HBPM) is often a convenient and practical alternative in usual care due to its ease to use [5, 6]. One of the crucial considerations for accurate HBPM is adhering to proper measurement techniques. The recommended posture for accurate BP measurement includes keeping the arm at heart level, legs uncrossed, and sitting with the back supported by a chair [1, 5]. These recommendations, provided in the guidelines, are formulated and presented with consideration for the cultural practice of using chairs in daily life. However, in Asian countries such as India, Japan, and Korea, the culture of sitting on the floor is as common or even more familiar than sitting on chairs at home [7–9]. For those accustomed to sitting on the floor in their daily lives, measuring BP while seated on the floor may be more convenient than sitting in a chair for HBPM. However, there is lack of research in BP measurements between seated in a chair and sitting on the floor can be interchangeable [10].

Therefore, the aim of the study was to evaluate whether there is a difference between BP measurements taken while sitting in a chair and while sitting on the floor in a Korean adult.

## Methods

### Study design

We requested the participation of consecutive subjects who are scheduled to undergo pulse wave velocity measurements for any reason at Hanyang University Medical Center between January and August 2023. The inclusion criteria were individuals aged 20 years or older who understood the research objectives and had provided written consent to participate in the study. The exclusion criteria were: individuals with a history of cognitive impairment, stroke, or mental illnesses; individuals with arteriovenous fistula or graft for hemodialysis; individuals with peripheral vascular disease in one or both arms; those unable to change position without assistance; individuals who smoked, consumed alcohol or caffeine, or engaged in exercise within 30 min prior to their visit; individuals with an arm circumference of less than 22 cm or greater than 32 cm; and individuals who declined to participate in the study. This study protocol was approved by the Institutional Review Board of the Hanyang University Medical Center, and a written informed consent was obtained from all participants.

### Data collection

The patients’ demographic and clinical characteristics, including age; sex; medical history of hypertension (and use of antihypertensive medication), diabetes, dyslipidemia, stroke, myocardial infarction, angina, congestive heart failure, and arrhythmia were obtained were obtained through questionnaires.

### Blood pressure measurement

**Fig. 1:**
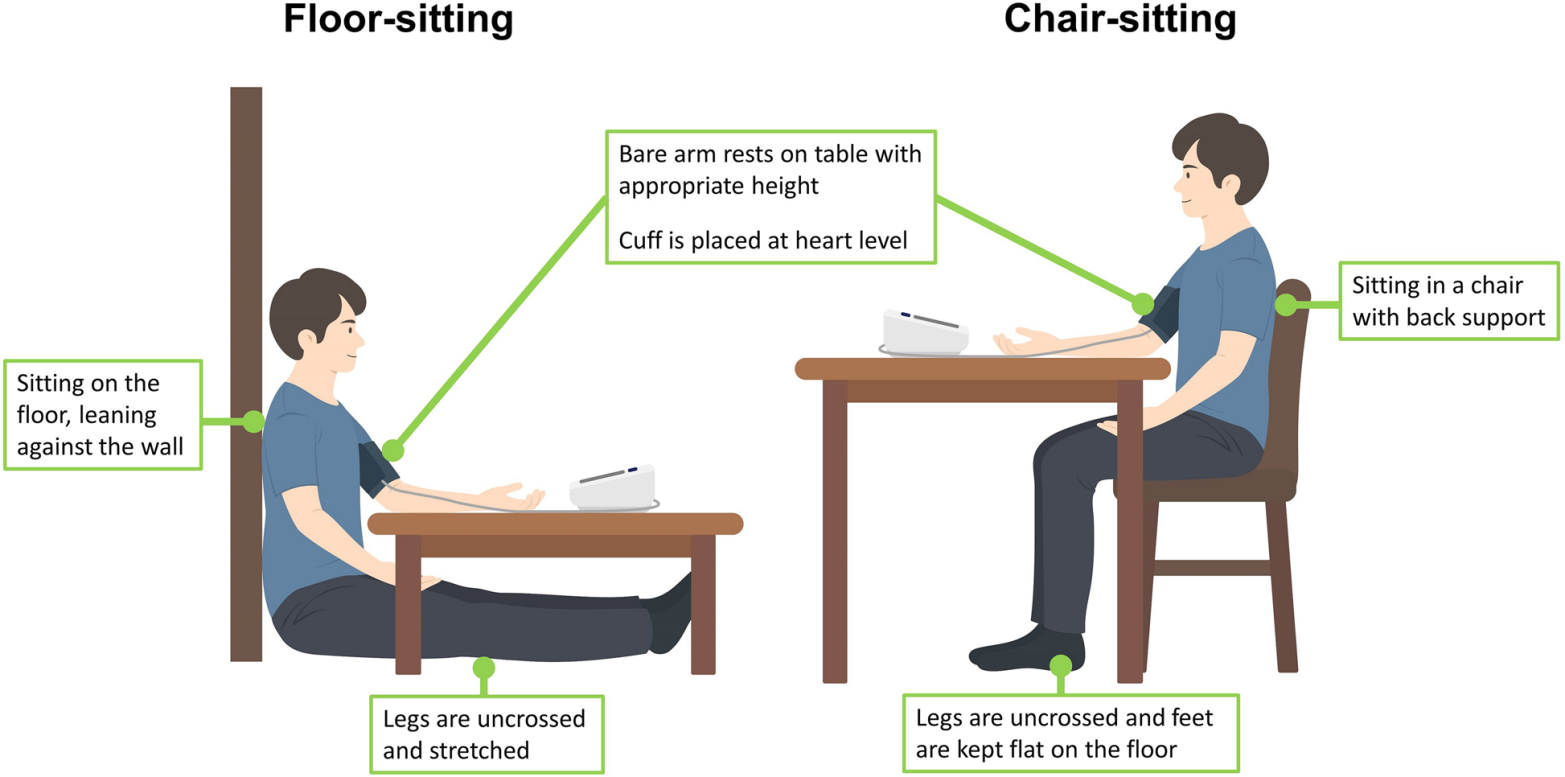
Two positions for blood pressure measurement

Participants were randomized in a non-blinded manner for the sequence of BP measurements posture using a coin toss. All measurement was carried out by the single observer. BP measurement posture was described in detail in Table S1 and Fig. 1. Before the examination, the patient rested in a quiet, temperature-controlled room for at least 5 min. If the initial measurement was in the chair-sitting position, participants rested by sitting on the chair with their back against the chair, legs uncrossed, and feet flat on the floor. If the initial measurement was in the floor-sitting position, participants rested by sitting on the floor, leaning against the wall with stretched legs. BP was measured in the right arm in both positions. After changing the BP measurement posture, BP was measured in the next posture after at least 1 min of rest. During the BP measurement, the upper arm cuff was maintained at heart level. BP was measured using a commercially available automated oscillometric device (Omron HEM-7080IC; Omron Healthcare, Kyoto, Japan) with an appropriately sized standard bladder for arm circumference, as indicated by the instruction manual.

### Statistical analyses

The main objective of this study was to determine whether there were clinically significant differences in BP measurements taken in two different postures. We arbitrarily considered a 2 mmHg mean BP difference to be clinically significant. Additionally, based on our previous research, we set the standard deviation (SD) of the repeated BP differences to the maximum value of 6 mmHg [11]. The sample size of 99 participants was calculated with a power of 0.95 and an alpha of 0.05, and we planned to conservatively recruit 120 participants.

Categorical variables were presented as frequencies with percentages, while continuous variables were reported as means with standard deviations or medians with interquartile ranges. The McNemar test was utilized for comparing categorical variables, paired t-tests for comparing normally distributed continuous variables, and the Wilcoxon signed-rank test for comparing continuous variables with a non-normal distribution. The distribution of continuous variables was assessed using the Shapiro–Wilk test. The Bland–Altman plot and intraclass correlation coefficients (ICC) was used to show agreement and absolute differences between two BP measurements. The ICC agreements were presented with 95% confidence intervals. Furthermore, a subgroup analysis was conducted using paired t-tests or Wilcoxon signed-rank tests, stratified by hypertension, systolic BP (SBP ≥ 140 or < 140 mmHg), diabetes, and sex. Statistical significance was defined as a two-sided *P*-value < 0.05. All statistical analyses were performed using the open-source statistical software R (version 4.3.1, www.R-project.org) and R-studio (version 2023.09.1 + 494, www.rstudio.com), along with relevant statistical packages such as tableone, blandr, irr, and ggplot2.

## Results

### General participant characteristics

A total of 116 participants were recruited. The median age of the patients was 68 (interquartile range, 59 to 75) years and 82 (70.7%) were men. The percentage of medical history of hypertension was 84.5%. There were 41 (35.3%) participants with diabetes, 103 (88.8%) participants with dyslipidemia, and 37 (31.9%) participants had histories of myocardial infarction (Table 1).

**Table 1:** Baseline characteristics

	All patients (*N* = 116)
Age (years)	68 (59, 75)
Male sex (%)	82 (70.7)
Comorbidities	
Hypertension (%)	98 (84.5)
Diabetes (%)	41 (35.3)
Dyslipidemia (%)	103 (88.8)
Stroke (%)	2 (1.7)
Myocardial infarction (%)	37 (31.9)
Angina (%)	31 (26.7)
Congestive heart failure (%)	3 (2.6)

Data are presented as *n* (%) or median (interquartile ranges), as appropriate

### Comparison of blood pressure measurements in two positions

**Fig. 2:**
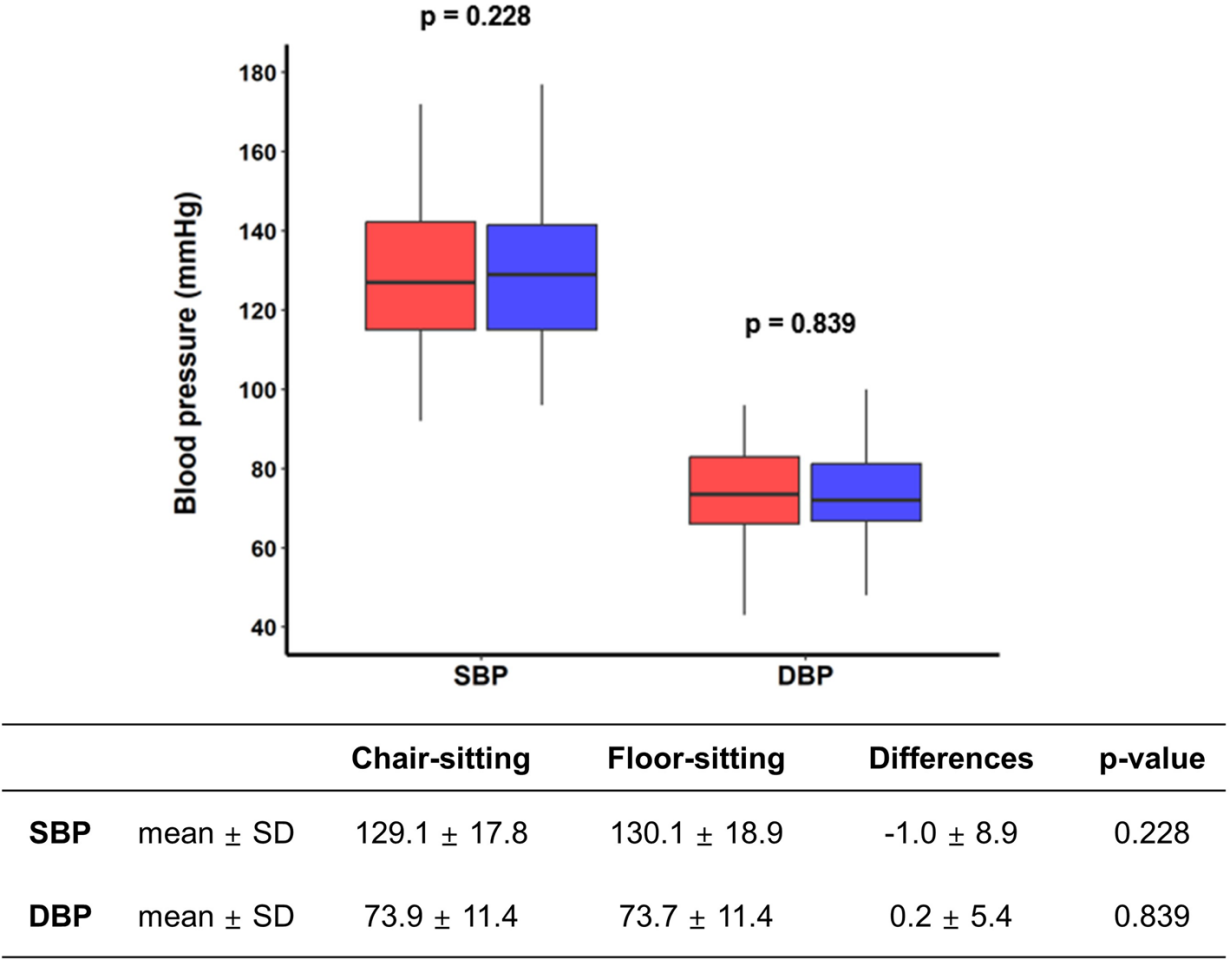
Comparison of blood pressure measurements between seated in a chair (chair-sitting) and sitting on the floor (floor-sitting). SBP, systolic blood pressure; DBP, diastolic blood pressure

The average SBPs and DBPs were 129.1 ± 17.8 mmHg and 73.9 ± 11.4 mmHg in chair-sitting and 130.1 ± 18.9 mmHg and 73.7 ± 11.4 mmHg in floor-sitting position. The absolute differences in BP measurements between the two positions were small (-1.0 ± 8.9 mmHg for SBP and 0.2 ± 5.4 mmHg for DBP), and were not statistically significant (*p* = 0.228 for SBP and *p* = 0.839 for DBP, Fig. 2).

**Fig. 3:**
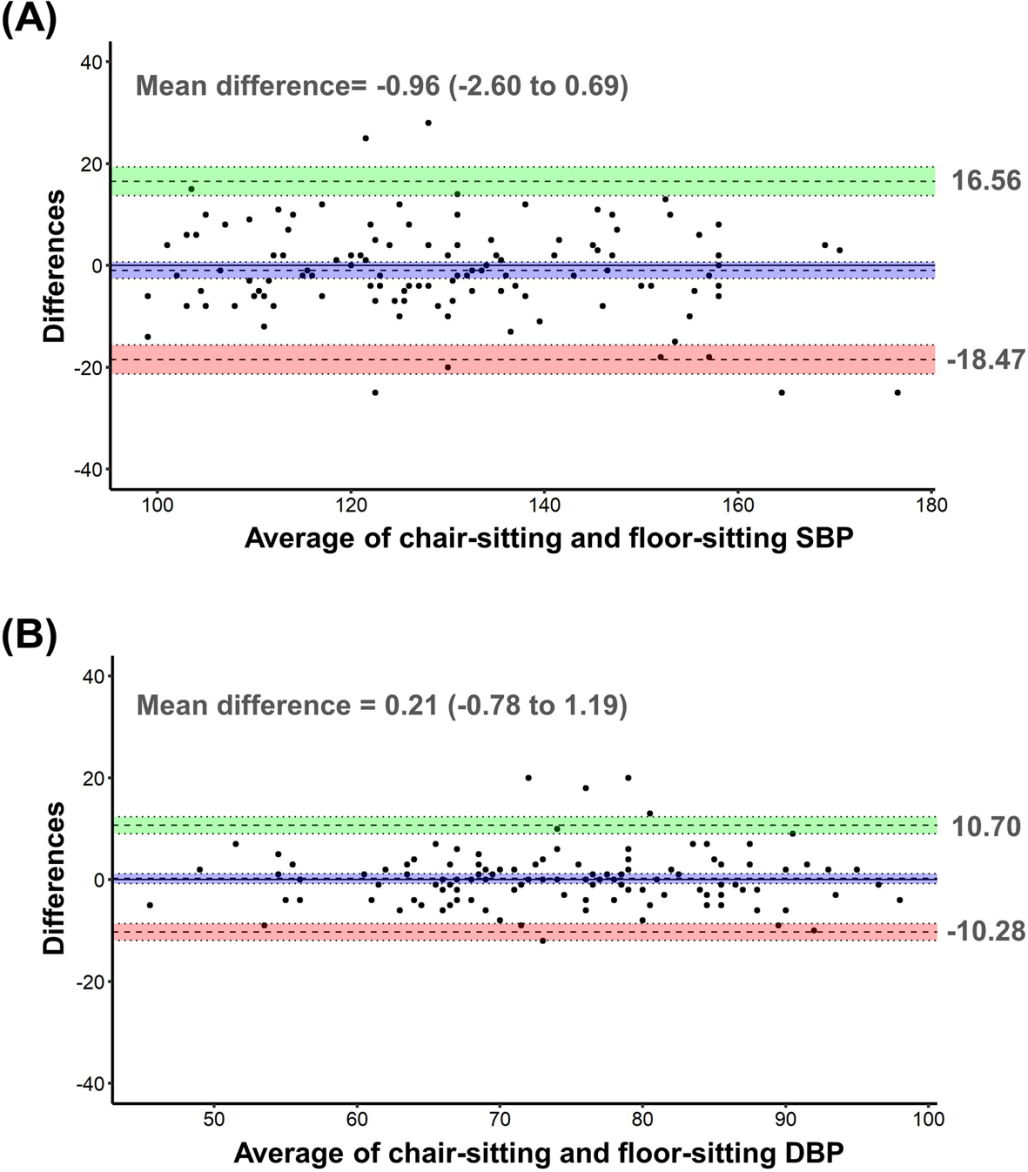
The Bland–Altman plots for agreement between two blood pressure measurement position. SBP, systolic blood pressure; DBP, diastolic blood pressure

The Bland–Altman plots for chair-sitting and floor-sitting showed that the differences of SBP and DBP were evenly distributed across the BP range, and the limits of agreement were from -18.47 to 16.56 mmHg for SBP and from -10.28 to 10.70 for DBP (Fig. 3). Table 2 showed the ICC agreements for BP measurements between the two positions. There was a very high level of agreement between BP measurements taken in the two positions (ICC = 0.882 for SBP and ICC = 0.890 for DBP).

**Table 2:** Intraclass correlations of agreement between chair-sitting and floor-sitting blood pressure

	Intraclass correlations	95% Confidence intervals
Chair-sitting versus floor-sitting SBP	0.882	0.834 to 0.916
Chair-sitting versus floor-sitting DBP	0.890	0.845 to 0.922

*SBP* systolic blood pressure, *DBP* diastolic blood pressure

### Subgroup analysis

**Fig. 4:**
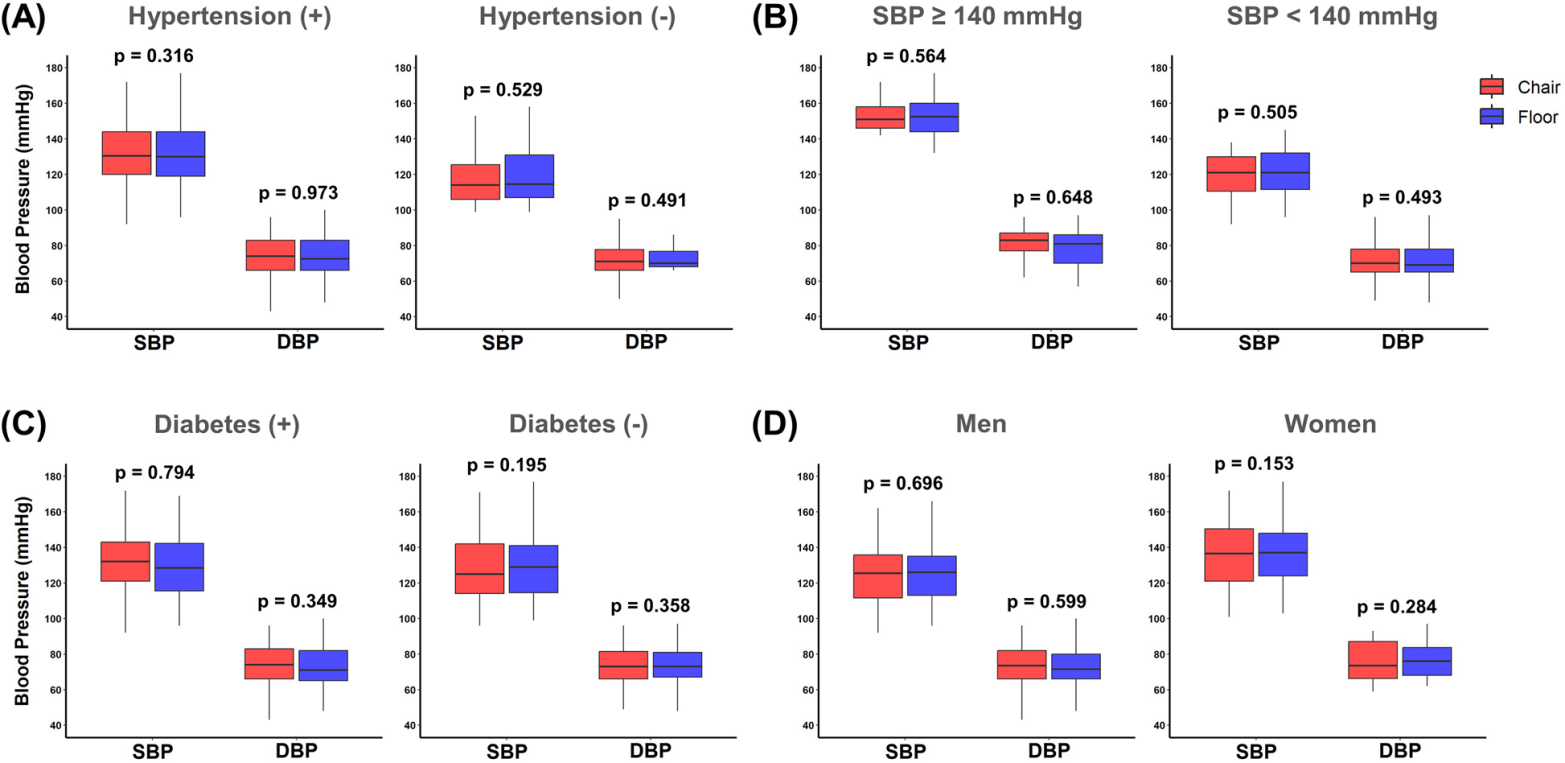
Comparison of blood pressure measurements between chair-Sitting and floor-sitting positions across subgroups based on **A** hypertension (yes or no), **B** systolic blood pressure (≥ 140 mmHg or < 140 mmHg), **C** diabetes (yes or no), and **D** sex (men or women)

In the subgroup analysis, we investigated the comparison of BP measurements between chair-sitting and floor-sitting positions based on various factors, including hypertension, SBP (≥ 140 or < 140 mmHg), diabetes, and sex. Participants with hypertension showed comparable SBP (130.5 vs. 131.0 mmHg, *p* = 0.316) and DBP (74.0 vs. 72.5 mmHg, *p* = 0.973) values in both positions. Among those without hypertension, no significant differences were observed in SBP (117.5 vs. 118.6 mmHg, *p* = 0.529) and DBP (71.0 vs. 70.0 mmHg, *p* = 0.491) between chair-sitting and floor-sitting positions. Individuals with SBP ≥ 140 mmHg and SBP < 140 mmHg showed comparable SBP (*p* = 0.564 in SBP ≥ 140 mmHg and *p* = 505 in SBP < 140 mmHg) and DBP (*p* = 0.648 in SBP ≥ 140 mmHg and *p* = 493 in SBP < 140 mmHg) values in both positions. Likewise, for participants with or without diabetes, as well as for males and females, no significant differences were observed in SBP and DBP between chair-sitting and floor-sitting positions (Fig. 4 and Table S2).

## Discussions

The aim of this study was to evaluate whether there are differences in BP measurements taken while sitting in a chair and sitting on the floor in Korean adults. This is the first evidence demonstrating whether measuring BP while sitting on the floor, a common practice in Asian cultures, is equivalent to the standard method of measuring BP while sitting in a chair. The key findings are as follows: 1) There were no significant differences in average SBP and DBP between the chair-sitting and floor-sitting positions; 2) ICC agreements indicated a very high level of agreement for BP measurements between the two positions; 3) SBP and DBP values between chair-sitting and floor-sitting positions were comparable across various subgroups, including those based on hypertension, SBP, diabetes, and sex.

Accurate BP measurement is essential for the diagnosis and treatment of hypertension [12]. Inadequate measurement methods can result in either overdiagnosis and unnecessary treatment or underdiagnosis, leading to an increased risk of preventable cardiovascular disease. Therefore, hypertension societies consistently publish literature on this subject, providing education and emphasizing the importance of precise BP measurement methods [1, 5, 13]. This standard BP measurement is crucial not only when measured in the office but also during HBPM. HBPM-based treatments are strongly recommended for hypertension control, as they provide greater reproducibility and outcome predictability compared to office BP [5, 14]. Additionally, they offer greater convenience, help avoid the white coat effect, and contribute to improved patient engagement with BP management [1, 15]. The prevalence of HBPM use is increasing, with global estimates for the prevalence of HBPM self-monitoring ranging between 30 and 70% [16–18]. However, it is estimated that many patients may not strictly adhere to BP self-measurement techniques recommended in guidelines [19, 20]. Various factors could be involved here [21, 22]. Especially for those who are accustomed to sitting on the floor, adhering to current guidelines for HBPM can be very challenging.

Numerous studies have identified various factors, including patient-related factors such as acute meal ingestion, acute alcohol use, acute caffeine use, acute nicotine use, bladder distension, and cold exposure, as well as device-related and procedure-related factors associated with measurement posture, that can impact the accuracy of BP measurement [23]. In relation to blood pressure measurement posture, it has been reported that differences in SBP of 3–10 mmHg can occur between supine and sitting positions, 5–15 mmHg between back/feet unsupported and supported positions, 1–7 mmHg between arm unsupported and supported positions, 5–8 mmHg between legs crossed and not crossed, and more than 10 mmHg between arm below heart level and arm at heart level positions [22]. The recommended BP measurement postures in the current guidelines have been established, taking these factors into account [1, 5, 13].

Considering the convenience, which is a significant advantage of HBPM, it is important to have a familiar and comfortable environment in terms of BP measurement posture. Evaluating the interchangeability of BP measurements based on seated positions is crucial for gathering evidence to formulate appropriate guidelines, especially for patients accustomed to a lifestyle that involves sitting on the floor—an important cultural consideration. Therefore, the results of this study have the strength of contributing to filling the gap in crucial evidence from the perspective of public health.

It is important to acknowledge some limitations of this study. Firstly, the single-center design may limit the generalizability of the results. Secondly, while efforts were made to adhere to standard BP measurement practices beyond seating posture, measurements were not conducted over several days. Additionally, since BP measurements were performed in a clinic setting due to methodological limitation, there may be differences compared to measurements taken at home. Lastly, as the need for home BP measurement is not limited to hypertensive patients, we recruited participants consecutively irrespective of their hypertension status or control. Consequently, a wide range of BP values was observed, leading to larger SD of BP differences and wider limits of agreement than anticipated. We believe these results reflect real-world clinical scenarios. Nonetheless, considering the small BP differences and high level of agreement, we anticipate that these results will not impede the interpretation of our primary findings significantly. To validate and build upon our findings, further investigation through larger, prospective, multicenter studies would be advantageous.

## Conclusions

In conclusion, this study contributes to our knowledge regarding the influence of seated positions on BP measurements, especially within the context of cultural practices. The findings indicate that chair-sitting and floor-sitting positions are interchangeable during BP measurement in Korean adults. This insight serves as a basis for refining guidelines and improving the accuracy of hypertension management practices, particularly in regions where floor-sitting is prevalent.

### Supplementary Information

Supplementary Material 1.

## Data Availability

The datasets used and/or analyzed during the current study are available from the corresponding author on reasonable request.

## Abbreviations

ABPM: Ambulatory blood pressure monitoring
BP: Blood pressure
DBP: Diastolic blood pressure
HBPM: Home blood pressure monitoring
ICC: Intraclass correlation coefficients
SBP: Systolic blood pressure
SD: Standard deviation
