# Supplementary material for: Comparing blood pressure measurements between sitting in chairs and sitting on the floor
Source: Clin Hypertens. 2024 Jul 1;30:16. doi: 10.1186/s40885-024-00273-w (PMC11215825; doi:10.1186/s40885-024-00273-w)
Supplement: Supplementary file 1 — Supplementary Material 1. [file 40885_2024_273_MOESM1_ESM.docx]

**Supplementary Table 1**. Method for blood pressure measurement

| **Process** | **Chair-sitting^†^** | **Floor-sitting^‡^** |
| --- | --- | --- |
| **Device and cuff** | Validated automated upper-arm device with proper cuff size according to the device’s instructions. | |
|  | Proper placement of cuff at the heart level.   - Placement of cuff on the mid-arm with the lower edge of the cuff 2–3 cm above the antecubital fossa. | |
| **Preparation** | No smoking, alcohol or caffeine, exercise, and bathing 30min before measurement. | |
|  | No talking during measurement and between measurements. | |
|  | Resting for 5min in a quiet room with comfortable temperature. | |
| **Position** | Sitting in a chair with back support. | Sitting on the floor, leaning against the wall. |
|  | Legs uncrossed and feet kept flat on the floor. | Legs uncrossed and stretched. |
|  | Bare arm resting on table and placement of cuff at the heart level. | Bare arm resting on low-height table and placement of cuff at the heart level. |

^†^The blood pressure of the chair-sitting was measured according to the home blood pressure measurement method proposed by the Korean Hypertension Society guideline [2].

^‡^All other conditions are the same as those for chair-sitting, except for the position.

| \| **Supplemental Table 2**. Comparison of blood pressure measurements between chair-sitting and floor-sitting positions across subgroups based on hypertension, systolic blood pressure, diabetes, and sex. \| \| \| \| \| --- \| --- \| --- \| --- \| \|  \| Chair-sitting \| Floor-sitting \| p-value \| \| \| Overall, N=116 \|  \|  \|  \| \| \| SBP \| 129.1 ± 17.8 \| 130.1 ± 18.9 \| 0.228 \| \| \| DBP \| 73.9 ± 11.4 \| 73.7 ± 11.4 \| 0.839 \| \| \| PP \| 55.2 ± 13.6 \| 56.4 ± 16.0 \| 0.092 \| \| \| Hypertension (yes), N=98 \|  \|  \|  \| \| \| SBP \| 130.5 (120.0, 144.0) \| 131.0 (119.0, 144.0) \| 0.316 \| \| \| DBP \| 74.0 (66.0, 83.0) \| 72.5 (66.0, 83.0) \| 0.973 \| \| \| PP \| 57.1 ± 13.4 \| 58.2 ± 15.9 \| 0.133 \| \| \| Hypertension (no), N=18 \|  \|  \|  \| \| \| SBP \| 117.5 ± 15.4 \| 118.6 ± 15.6 \| 0.529 \| \| \| DBP \| 71.0 (66.0, 77.8) \| 70.0 (68.0, 76.8) \| 0.491 \| \| \| PP \| 45.1 ± 10.3 \| 46.3 ± 13.0 \| 0.434 \| \| \| SBP ≥140 mmHg, N=33 \|  \|  \|  \| \| \| SBP \| 152.0 ± 8.0 \| 152.5 ± 13.7 \| 0.794 \| \| \| DBP \| 83.0 (77.0, 87.0) \| 81.0 (70.0, 86.0) \| 0.349 \| \| \| PP \| 70.9 ± 9.5 \| 72.9 ± 13.1 \| 0.238 \| \| \| SBP ≥140 mmHg, N=83 \|  \|  \|  \| \| \| SBP \| 120.0 ± 11.2 \| 121.2 ± 12.1 \| 0.195 \| \| \| DBP \| 70.0 (65.0, 78.0) \| 69.0 (65.0, 78.0) \| 0.358 \| \| \| PP \| 49.0 ± 9.4 \| 49.8 ± 11.8 \| 0.235 \| \| \| Diabetes (yes), N=41 \|  \|  \|  \| \| \| SBP \| 131.2 ± 18.0 \| 132.1 ± 20.9 \| 0.564 \| \| \| DBP \| 74.0 (66.0, 83.0) \| 71.0 (65.0, 82.0) \| 0.648 \| \| \| PP \| 57.1 ± 15.7 \| 59.1 ± 18.2 \| 0.117 \| \| \| Diabetes (no), N=75 \|  \|  \|  \| \| \| SBP \| 125.0 (114.0, 142.0) \| 129.0 (114.5, 141.0) \| 0.505 \| \| \| DBP \| 73.0 (66.0, 81.5) \| 73.0 (67.0, 81.0) \| 0.493 \| \| \| PP \| 53.0 (43.0, 62.0) \| 55.0 (43.5, 63.5) \| 0.718 \| \| \| Men, N=82 \|  \|  \|  \| \| \| SBP \| 126.0 ± 16.6 \| 126.4 ± 17.2 \| 0.696 \| \| \| DBP \| 73.5 (66.0, 82.0) \| 71.5 (66.0, 80.0) \| 0.599 \| \| \| PP \| 52.9 ± 13.5 \| 53.9 ± 16.0 \| 0.231 \| \| \| Women, N=34 \|  \|  \|  \| \| \| SBP \| 136.5 ± 18.8 \| 138.9 ± 20.2 \| 0.153 \| \| \| DBP \| 73.5 (66.3, 87.0) \| 76.0 (68.0, 83.8) \| 0.284 \| \| \| PP \| 60.7 ± 12.4 \| 62.4 ± 14.7 \| 0.232 \| \| |
| --- | --- | --- | --- | --- | --- | --- | --- | --- | --- | --- | --- | --- | --- | --- | --- | --- | --- | --- | --- | --- | --- | --- | --- | --- | --- | --- | --- | --- | --- | --- | --- | --- | --- | --- | --- | --- | --- | --- | --- | --- | --- | --- | --- | --- | --- | --- | --- | --- | --- | --- | --- | --- | --- | --- | --- | --- | --- | --- | --- | --- | --- | --- | --- | --- | --- | --- | --- | --- | --- | --- | --- | --- | --- | --- | --- | --- | --- | --- | --- | --- | --- | --- | --- | --- | --- | --- | --- | --- | --- | --- | --- | --- | --- | --- | --- | --- | --- | --- | --- | --- | --- | --- | --- | --- | --- | --- | --- | --- | --- | --- | --- | --- | --- | --- | --- | --- | --- | --- | --- | --- | --- | --- | --- | --- | --- | --- | --- | --- | --- | --- | --- | --- | --- | --- | --- | --- | --- | --- | --- | --- | --- | --- | --- | --- | --- | --- | --- | --- | --- | --- | --- | --- | --- | --- | --- | --- | --- | --- | --- | --- | --- | --- | --- | --- | --- | --- | --- | --- | --- | --- | --- | --- | --- | --- | --- | --- | --- | --- | --- | --- | --- | --- | --- | --- | --- | --- | --- | --- | --- |

BP, blood pressure, SBP, systolic blood pressure; DBP, diastolic blood pressure; PP, pulse pressure.
